# Loss of β2-integrin function results in metabolic reprogramming of dendritic cells, leading to increased dendritic cell functionality and anti-tumor responses

**DOI:** 10.1080/2162402X.2024.2369373

**Published:** 2024-06-21

**Authors:** Heidi Harjunpää, Riku Somermäki, Guillem Saldo Rubio, Manlio Fusciello, Sara Feola, Imrul Faisal, Anni I Nieminen, Liang Wang, Marc Llort Asens, Hongxia Zhao, Ove Eriksson, Vincenzo Cerullo, Susanna C Fagerholm

**Affiliations:** a Molecular and Integrative Biosciences Research Programme, Faculty of Biological and Environmental Sciences, University of Helsinki, Helsinki, Finland; b Faculty of Pharmacy, University of Helsinki, Helsinki, Finland; c Institute for Molecular Medicine Finland (FIMM), University of Helsinki, Helsinki, Finland; d Biochemistry and Developmental biology, Medicum, Faculty of Medicine, University of Helsinki, Helsinki, Finland

**Keywords:** Integrin, dendritic cell, cancer immunotherapy, cell metabolism, cell adhesion, CCR7, glycolysis, 2-deoxyglucose, Ikaros, mTOR

## Abstract

Dendritic cells (DCs) are the main antigen presenting cells of the immune system and are essential for anti-tumor responses. DC-based immunotherapies are used in cancer treatment, but their functionality is not optimized and their clinical efficacy is currently limited. Approaches to improve DC functionality in anti-tumor immunity are therefore required. We have previously shown that the loss of β2-integrin-mediated adhesion leads to epigenetic reprogramming of bone marrow-derived DCs (BM-DCs), resulting in an increased expression of costimulatory markers (CD86, CD80, and CD40), cytokines (IL-12) and the chemokine receptor CCR7. We now show that the loss of β2-integrin-mediated adhesion of BM-DCs also leads to a generally suppressed metabolic profile, with reduced metabolic rate, decreased ROS production, and lowered glucose uptake in cells. The mRNA levels of glycolytic enzymes and glucose transporters were reduced, indicating transcriptional regulation of the metabolic phenotype. Surprisingly, although signaling through a central regulator of immune cell metabolisms, the mechanistic target of rapamycin (mTOR), was increased in BM-DCs with dysfunctional integrins, rapamycin treatment revealed that mTOR signaling was not involved in suppressing DC metabolism. Instead, bioinformatics and functional analyses showed that the Ikaros transcription factor may be involved in regulating the metabolic profile of non-adhesive DCs. Inversely, we found that induction of metabolic stress through treatment of cells with low levels of an inhibitor of glycolysis, 2-deoxyglucose (2DG), led to increased BM-DC activation. Specifically, 2DG treatment led to increased levels of *Il-12* and *Ccr7* mRNA, increased production of IL-12, increased levels of cell surface CCR7 and increased *in vitro* migration and T cell activation potential. Furthermore, 2DG treatment led to increased histone methylation in cells (H3K4me3, H3K27me3), indicating metabolic reprogramming. Finally, metabolic stress induced by 2DG treatment led to improved BM-DC-mediated anti-tumor responses *in vivo* in a melanoma cancer model, B16-OVA. In conclusion, our results indicate a role for β2-integrin-mediated adhesion in regulating a novel type of metabolic reprogramming of DCs and DC-mediated anti-tumor responses, which may be targeted to enhance DC-mediated anti-tumor responses in cancer immunotherapy.

## Introduction

DCs are the main antigen presenting cells of the immune system and play major roles in activating and polarizing the immune response. In peripheral tissues, DCs first take up antigens followed by activation (maturation and reprogramming) in response to associated pathogen and danger signals and then migrate to lymph nodes where they activate and polarize T cell responses.^1^ Because of their essential role in T cell activation, DCs are also used in immunotherapeutic approaches to treat cancer. Currently, the only FDA-approved DC-based vaccine (for prostate cancer) is Provenge, which consists of monocyte-derived DCs which are expanded from patients’ peripheral blood, loaded with tumor antigens and injected back into patients. However, although they are safe and well tolerated, the clinical efficacy of dendritic cell vaccines is limited, at least in part due to poor DC migration to lymph nodes and/or due to limited T cell activation/polarization capacity.^2,3^ Therefore, understanding how to improve DC functionality *in vitro* and *in vivo* could offer new approaches to enhance immunotherapy for cancer, either alone or in combination with other therapies.

DC activation is associated with large changes in gene expression as well as epigenetic changes (reprogramming).^4^ Simultaneously, the cells undergo fundamental changes in integrin-mediated adhesiveness and phagocytic capacity, which are both downregulated when the cells acquire the migratory phenotype.^5–7^ DC activation is also associated with metabolic changes such as reduced oxidative phosphorylation, increased aerobic glycolysis, and changes in lipid metabolism.^8,9^ The mechanistic target of rapamycin (mTOR) signaling pathway is often involved in metabolic reprogramming of immune cells and has previously been reported to be important also for TLR-induced DC metabolic reprogramming.^10^ In particular, mTOR/HIF-1α/iNOS signaling is required for sustained glycolytic reprogramming at later time points after activation, which is required for DC survival.^8^

Integrins are pivotal adhesion and mechanoreceptors in cells, which play a central role in immune cell trafficking and effector functions. β2-integrins are leukocyte-specific molecules that play diverse roles in DCs, including cDC2 positioning in spleen^11^ and trafficking of DCs to lymph nodes in inflammatory but not homeostatic conditions.^1^ Interestingly, β2-integrins can also restrict immune cell responses by, e.g., inhibiting toll-like receptor signaling in macrophages and DCs, promoting tolerance, and suppressing colitis and dermatitis.^12–17^ Thus, β2-integrins can also play homeostatic and regulatory roles in immunity and inflammation. We have previously shown that β2-integrins inhibit bone marrow-derived DC (BM-DC) activation/reprogramming *in vitro* and BM-DC-mediated Th1 polarization and anti-tumor responses *in vivo*.^6,14,18^ In TTT/AAA-β2-integrin knock-in mice (β2-integrin KI), where β2-integrins are expressed but dysfunctional due to reduced binding to kindlin-3, an important cytoplasmic regulator of integrins, there are increased numbers of migratory DCs (migDCs) in lymph nodes and spleen. In addition, endogenous β2-integrin KI DCs display increased trafficking to lymph nodes and increased maturation with elevated expression of CD86 *in vivo*.^7^ Increased BM-DC activation in response to loss of integrin-mediated adhesion occurs through changes in epigenetic (e.g. histone methylation, H3K4me3, and H3K27me3) and chromatin landscapes, large changes in gene expression (increased expression of, e.g., *Cd86*, *Cd40*, *Ccr7*, *Fscn1*, and *Il12*), and fundamental changes in transcription factors of Ikaros and RelA-mediated gene expression.^18,19^ Integrins thus mediate interactions between the DC and its microenvironment, hence suppressing DC reprogramming and maturation both *in vitro* and *in vivo* settings, contributing to immune homeostasis.

As myeloid cell reprogramming is well known to be associated both with epigenetic and with metabolic changes,^8,9,20^ here, we investigated whether integrin dysfunction is also associated with metabolic changes in DCs, using BM-DCs as a model system. Our results reveal major metabolic reprogramming in BM-DCs expressing dysfunctional β2-integrins with reduced metabolism, and production of reactive oxygen species (ROS). The expression of glycolytic enzymes and glucose transporters was downregulated, and the metabolic reprogramming was shown to be partially driven by the transcription factor Ikaros. We also show that mimicking such metabolic stress in wild-type (WT) BM-DCs by treating cells with low concentration of glycolysis inhibitor, 2-deoxyglucose (2DG), drives increased DC activation (increased expression of IL-12 and CCR7, *in vitro* migration and T cell activation potential), increased H3K4me3 and H3K27me3 histone methylation, and enhanced anti-tumor responses *in vivo*. In conclusion, integrin-mediated cell adhesion regulates the metabolic and epigenetic phenotypes of BM-DCs, showing that the β2-integrin-mediated regulation of metabolic profile of DCs plays a pivotal role in their activation, which may be targeted to optimize anti-tumor responses of these cells.

## Results

### Inactivating β2-integrins leads to an altered metabolic profile of BM-DCs

**Figure 1.**
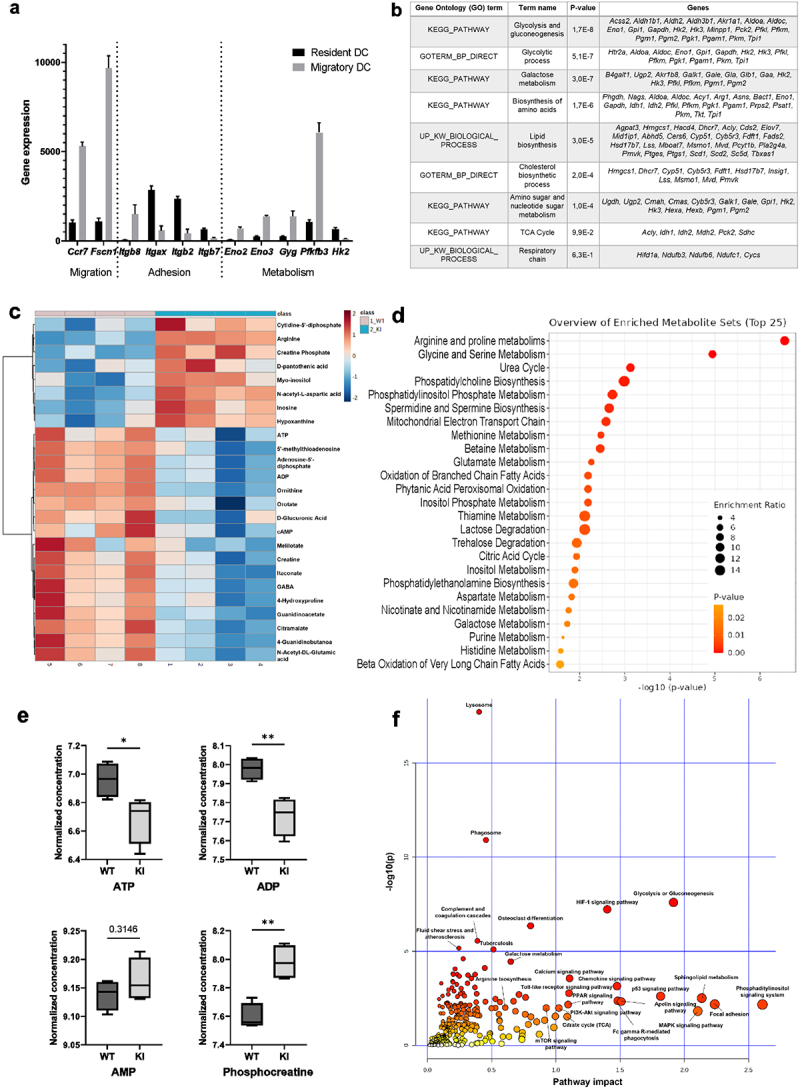
Multiomics analysis reveals an altered metabolic profile in BM-DCs expressing dysfunctional β2-integrins (a) Analysis of publicly available gene expression data of resident and migDCs^6^ for genes involved in cell migration, adhesion and cellular metabolism. (b) Analysis of downregulated pathways in previously published RNA-Seq data from β2-integrin KI BM-DCs^7^ using the DAVID bioinformatics tool. (c) Targeted mass spectrometry-based analysis of metabolites in WT and β2-integrin KI BM-DCs was performed as described in the Materials and methods section (n=4). (d) Metabolite set enrichment analysis (MSEA) of metabolites identified in WT and β2-integrin KI BM-DCs using the publicly available MetaboAnalyst platform. e) Levels of central metabolites identified by mass spectrometry (normalized concentration e.g. log10). (f) Integrated RNA-Seq and metabolomics data analysis to identify most highly affected metabolic pathways was performed using the MetaboAnalyst platform. P-values are shown as <0.05*, <0.01**.

As immune cell reprogramming often goes hand-in-hand with metabolic changes, we decided to investigate a putative relationship between integrin adhesion and cellular metabolism in DCs. We first investigated publicly available transcriptomics data of migDCs isolated from murine lymphoid organs for their expression levels of migratory, adhesion, and metabolic genes.^6^ Compared to tissue resident DCs, migDCs express higher mRNA levels of migratory genes such as those encoding for chemokine receptors *(Ccr7*) and actin-binding proteins associated with cell migration (*Fscn1*) (Figure 1a). In contrast, pathways such as “cell–cell adhesion” and “integrin signaling” were downregulated, including expression of *Itgax, Itgb2*, and *Itgb7* genes, encoding for αX-integrin, β2-integrin, and β7-integrin, respectively (Figure 1a). Interestingly, also changes in the expression of metabolic genes were detected. In particular, the cells express very low levels of *Hk2*, encoding for the rate-limiting enzyme of glycolysis (hexokinase 2). Instead, *Eno2, Eno3* (enolase, involved in glycolysis), and *Gyg* (glycogenin, involved in the synthesis of glycogen) genes were upregulated (Figure 1a). MigDCs *in vivo* therefore display changes in the expression of genes associated with both cell adhesion and glucose metabolism.

Based on their transcriptional profile, granulocyte-macrophage colony-stimulating factor (GM-CSF)-cultured murine BM-DCs resemble *in vivo* migDCs, making them a useful *in vitro* tool to investigate migDC function.^21^ We have previously shown that BM-DCs expressing dysfunctional β2-integrins display increased activation and migration and have extensively reported the phenotype of these cells (increased expression of CD80, CD86, CD40, CCR7, increased IL-12 production, increased 3D migration).^7,18^ Representative histograms of expression of DC markers in WT and KI BM-DCs are also shown in Supplementary SFig 1a. We here turned to investigate the metabolic status of BM-DCs expressing dysfunctional β2-integrins. Analysis of our previously published RNA-Seq data from WT and β2-integrin KI BM-DCs^7^ indeed indicated that several metabolic pathways were downregulated in the β2-integrin KI cells, including “glycolysis and gluconeogenesis”, “biosynthesis of amino acids” and “lipid biosynthesis” (Figure 1b), indicating a changed metabolic profile of BM-DCs expressing dysfunctional integrins. Thus, we further analyzed the metabolomes of WT and β2-integrin KI BM-DCs by targeted mass spectrometry metabolomics profiling analysis (Figure 1c). This analysis revealed changes in several metabolites in BM-DCs expressing dysfunctional integrins, including several metabolites in pathways such as “arginine and proline metabolism”, “urea cycle”, “glycine and serine metabolism”, and “mitochondrial electron transport” (Figure 1c–e). For example, the arginine levels were significantly increased, whilst itaconate, ornithine, orotate, and guanidinoacetate were decreased (Figure 1c). Analysis of RNA-Seq data confirmed significant downregulation of *Arg1*, the first enzyme in the arginine metabolic pathway, putatively explaining why especially arginine metabolism and the urea cycle were severely affected in these cells.^7^ Mass spectrometry analysis also revealed differences in ATP, ADP (but not AMP), and phosphocreatine (a metabolic energy store) levels between WT and β2-integrin KI cells, indicating a significant change in metabolic status as a result of integrin dysfunction (Figure 1e). Indeed, integrating metabolomics and RNA-Seq data indicated “glycolysis or gluconeogenesis” to be the pathway showing the highest impact and significance by the integrin mutation in BM-DCs (Figure 1f). Other pathways that may be affected include the HIF1 pathway and the phosphatidyl inositol signaling system, which are pathways previously implicated in the regulation of metabolic reprogramming of DCs.^22^ Therefore, our multi-omics data suggested changes in several metabolic pathways and in the metabolic status of BM-DCs expressing dysfunctional integrins.

### Reduced metabolic rate and altered mitochondrial function in BM-DCs expressing inactive β2-integrins

**Figure 2.**
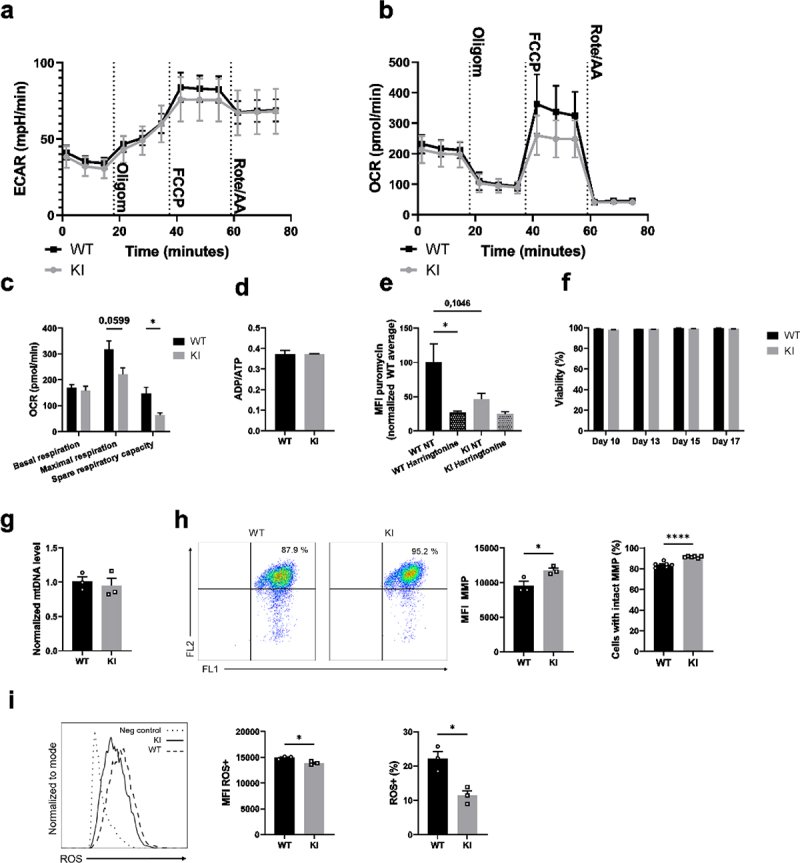
β2-integrin deficient BM-DCs display altered metabolic profile compared to BM-DCs with functional integrins. (a) Extracellular acidification rate (ECAR) and (b) oxygen consumption rate (OCR) of WT and β2-integrin KI BM-DCs were measured real time by utilizing Seahorse Extracellular Analyser and Mito Stress Test Kit (n=4). (c) Parameters indicating mitochondrial function in WT and β2-integrin KI BM-DCs were calculated based on OCR data shown in B). (d) ATP/ADP ratio in WT and β2-integrin KI BM-DCs was determined with luminescence-based ADP/ATP Ratio Assay Kit (n=3). (e) Metabolic activity of both WT and β2-integrin KI BM-DCs was assessed flow cytometrically by determining the level of puromycin incorporation. Harringtonine (translation inhibitor)-treated cells were used as a negative control and the result is normalized to the metabolic activity of WT cells. (n=3). Cell viability was assessed flow cytometrically (n=3). (g) Level of mitochondrial DNA (mtDNA) in WT and β2-integrin KI BM-DCs was assessed by qRT-PCR (n=3). (h) Mitochondrial membrane potential (MMP) and H) the level of reactive oxygen species (ROS) in WT and β2-integrin KI BM-DCs were assessed by flow cytometer with representative dot plots (h) or histograms (i), MFIs and proportions of positive cells shown (n=3). Data in a) and b) is representative of two independent experiments. Data in h) is either shown as representative of two independent experiments (dot plots and MFI) or pooled from the two independent experiments (proportions of positive cells). P-values are shown as <0.05*, <0.0001****.

Total cellular ATP is mainly generated by glycolysis and oxidative phosphorylation in mammalian cells. We found that the expression of glycolytic enzymes and ATP and ADP levels were reduced in β2-integrin KI BM-DCs (Figure 1e). In addition, glycolysis was heavily implicated as being regulated by integrins in the integrated multi-omics analysis (Figure 1f). To directly investigate the effect of integrin dysfunction on cellular metabolism, we turned to Seahorse Extracellular Flux analysis, capable of measuring extracellular acidification rate (ECAR) and oxygen consumption rate (OCR), thus indicating levels of glycolysis and oxidative phosphorylation, respectively, in cells in real time. Interestingly, we found differences in both ECAR (Figure 2a) and OCR (Figure 2b) between β2-integrin KI and WT BM-DCs. The basal rate of glycolysis (ECAR before injection of any cellular respiratory modulators) was not reduced in β2-integrin KI BM-DCs (Figure 2a). However, driving mitochondrial uncoupling with FCCP (which uncouples oxidative phosphorylation from ATP production, thereby driving increased aerobic glycolysis to compensate for the loss of ATP production; reflects cellular response to conditions of increased energy demand or stress) led to lower ECAR in integrin deficiency compared to WT cells (Figure 2a). In addition, BM-DCs expressing dysfunctional integrins displayed reduced maximal respiration, as well as spare respiratory capacity (which reflects the cell’s ability to respond to increased energy demand) (Figure 2b–c and Supplementary Fig S1b). However, basal respiration, proton leak, mitochondrial ATP production, or coupling efficiency were not affected (Figure 2b–c, Supplementary Fig S1b). In addition, although our mass spectrometry analysis indicated that ATP and ADP levels were reduced in BM-DCs expressing dysfunctional integrins (Figure 1c), the ATP/ADP ratio in cells was not affected by integrin dysfunction (Figure 2d, Supplementary SFig 1c).

The reduced metabolic rate of the β2-integrin KI BM-DCs compared to WT was confirmed by measuring protein translation as a proxy for available cellular energy. Protein translation has previously been shown to correlate with metabolic activity in lymphocytes.^23^ Indeed, the β2-integrin KI BM-DCs exhibited markedly reduced translation (puromycin incorporation) compared to WT BM-DCs (Figure 2e), confirming the lower metabolic rate in these cells.

Given that the overall metabolic status was decreased (lower level of protein translation and ATP) in β2-integrin KI compared to WT BM-DCs, we first assessed whether there was a difference in viability. No differences were observed between β2-integrin KI and WT BM-DCs even after 17 d in culture (Figure 2f). Next, we turned to investigate mitochondrial function in more detail. Firstly, we found that mitochondrial numbers were not affected (Figure 2g) in BM-DCs expressing dysfunctional β2-integrins. However, the mitochondrial membrane potential was slightly increased in β2-integrin KI BM-DCs (Figure 2h). In contrast, ROS levels were markedly reduced in cells with dysfunctional integrins (Figure 2i). Together with a reduced metabolism (Figure 2a–c) and lower translation rates (Figure 2e), these data indicated reduced glucose metabolism and mitochondrial function in β2-integrin KI BM-DCs.

### Suppressed metabolism is regulated on the transcriptional level in BM-DCs expressing dysfunctional integrins

**Figure 3.**
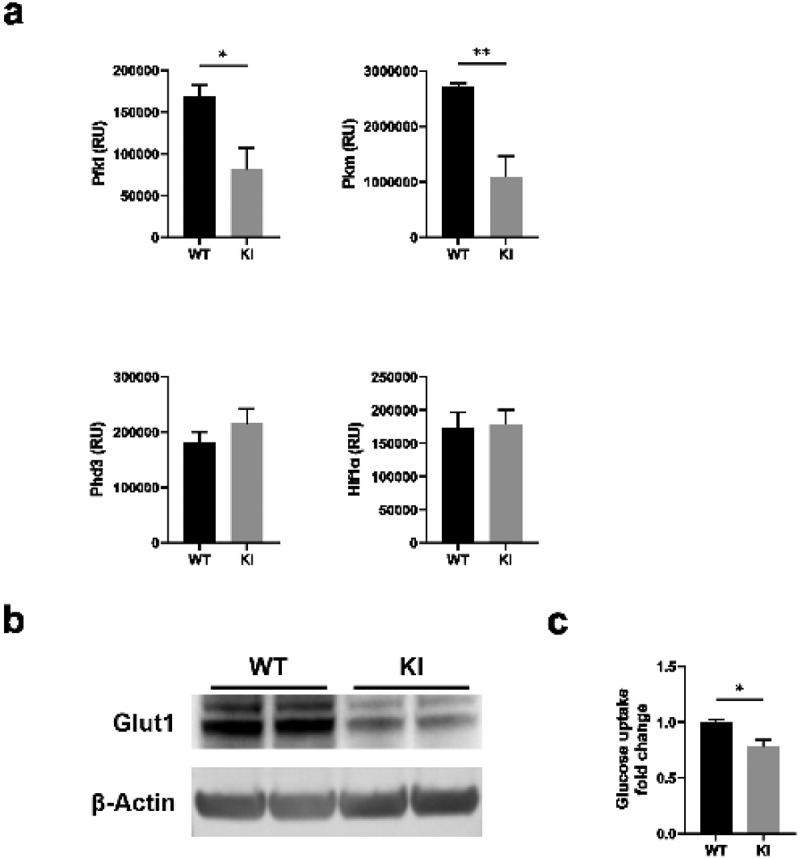
β2-integrin-deficient BM-DCs display reduced expression of glycolytic enzymes and glucose transporters as well as reduced glucose uptake. (a) Expression of Pfkl, Pkm, Hif1α and Phd3, b) protein level of Glut1 and c) glucose uptake in WT and β2-integrin KI BM-DC were determined by using a) qRT-PCR (n=3–5), (b) Western blotting (n=5) or (c) luminescence-based Glucose Uptake-Glo™ Assay kit (n=4) as described in the Materials and Methods. P-values are shown as <0.05*, <0.01**.

RNA-Seq analysis indicated that several glycolytic and glucose transporter genes were downregulated in BM-DCs expressing dysfunctional β2-integrins (Figure 1b). In addition, as there were differences in OCR rates under conditions of cellular stress, we next investigated the expression of genes involved in the pathways in WT and β2-integrin KI BM-DCs by qRT-PCR. Indeed, we found that the expression of glycolytic enzymes *Pfkl* and *Pkm* was significantly reduced in β2-integrin KI cells (Figure 3a). In contrast, there was no difference in the expression of genes such as *Hif1α* or *Phd3* (Figure 3a). In addition, we found that Glut1 was significantly downregulated at the protein level in these cells (Figure 3b). We therefore investigated glucose uptake in β2-integrin KI cells and found a significant downregulation of glucose uptake in these cells (Figure 3c). Together, these integrated multiomics data (Figures 1–3) showed a significant suppression of glycolytic metabolism of BM-DCs expressing dysfunctional β2-integrins. Furthermore, the data indicated that the altered metabolic profile is regulated, at least in part, on the transcriptional level.

### The mTOR pathway is not responsible for downregulation of glycolysis or oxidative phosphorylation in β2-integrin-deficient BM-DCs

**Figure 4.**
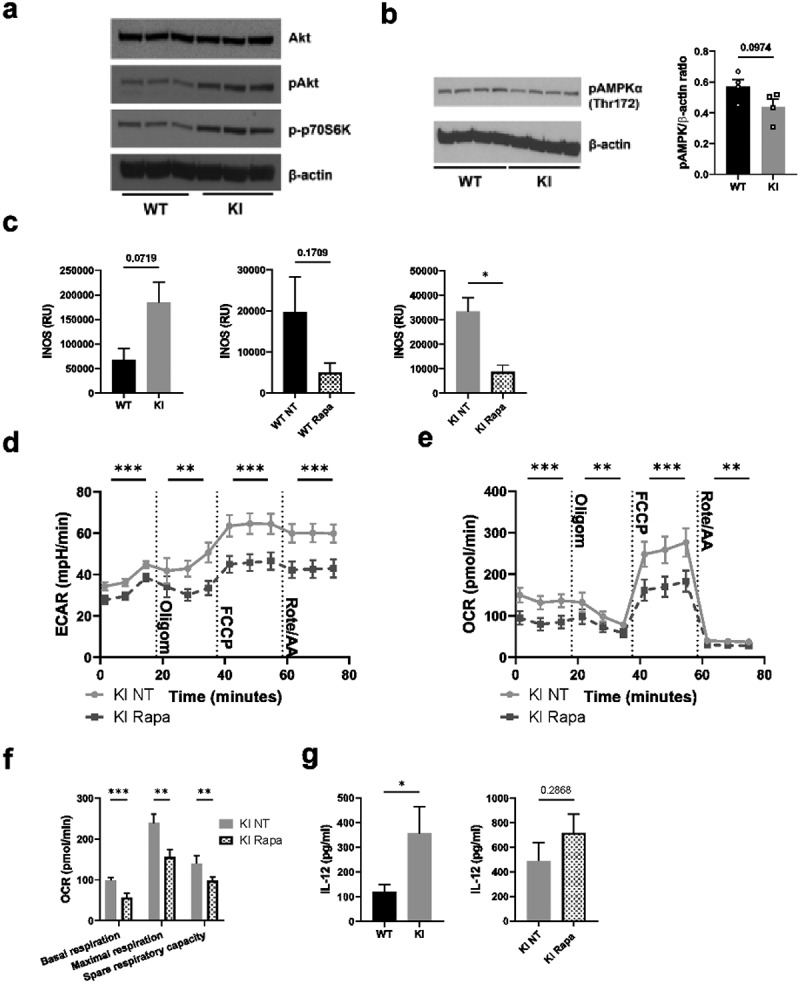
mTOR pathway is not responsible for the altered metabolic profile of β2-integrin KI BM-DCs. The activity of a) mTOR and b) AMPK pathways in WT and β2-integrin KI BM-DCs were assessed by Western blotting of (a) pAkt, phopho-p70S6K and b) pAMPKα (Thr172) (n=3–4). (b) for pAMPKα (Thr172), both the blot and quantification of the result are shown. (c) iNOS expression in WT and β2-integrin KI BM-DCs was determined by qRT-PCR in the presence or absence of rapamycin (n=3). (d) ECAR and e) OCR in β2-integrin KI BM-DCs, in the presence or absence of rapamycin, were analysed by Seahorse Extracellular analyser with a Mito Stress Test Kit (n=4) and (f) parameters of mitochondrial respiration were calculated from rates of oxygen consumption shown in (e). (g) ELISA was used to assess IL-12 production in WT and β2-integrin KI BM-DCs in the presence or absence of rapamycin. Data in g) is pooled from six (WT vs. KI) or four (KI vs. KI rapa) independent experiments. p-values are shown as <0.05*, <0.01**, <0.005***.

We next turned to investigate putative pathways that may regulate the metabolic phenotype of BM-DCs where integrins are dysfunctional. Here, we found that Akt and mTOR signaling were upregulated in cells expressing dysfunctional β2-integrins (Figure 4a), and there was a trend toward increased iNOS expression in these cells, although the results did not reach statistical significance (Figure 4c). The AMPK (AMP-activated protein kinase) pathway opposes mTOR, and responds to energy stress in cells (high AMP/ATP ratio, high creatine/phosphocreatine ratio).^24^ The activity of AMPK was slightly downregulated in β2-integrin KI cells (Figure 4b), possibly reflecting the high phosphocreatine levels in these cells (Figure 1b). Furthermore, the expression of iNOS was suppressed in β2-integrin KI cells by treatment with mTOR inhibitor, rapamycin (Figure 4c). However, we found that mTOR was not responsible for the differences in metabolism between WT and β2-integrin KI BM-DC, as treatment of β2-integrin KI cells with rapamycin did not reverse their metabolic phenotype (Figure 4d,e). Rather, rapamycin treatment further reduced ECAR and OCR rates and thus the overall metabolic rate in cells expressing dysfunctional integrins (Figure 4d–f, Supplementary Fig S1d). Both basal and maximal respiration were further reduced by rapamycin treatment, which also affected ATP production and spare respiratory capacity in β2-integrin KI BM-DCs (Figure 4f). In addition, as previously reported,^7^ as part of their activated phenotype, IL-12 production was increased in β2-integrin KI cells (Figure 4g). However, treatment of β2-integrin KI cells with rapamycin did not reduce IL-12 production in these cells (Figure 4g). Together, our data suggest that although mTOR signaling is upregulated in BM-DCs expressing dysfunctional integrins, mTOR signaling is not responsible for their suppressed metabolic state or their increased activation.

### Transcription factor Ikaros plays an important role in mediating the β2-integrin-regulated metabolic phenotype of BM-DCs

**Figure 5.**
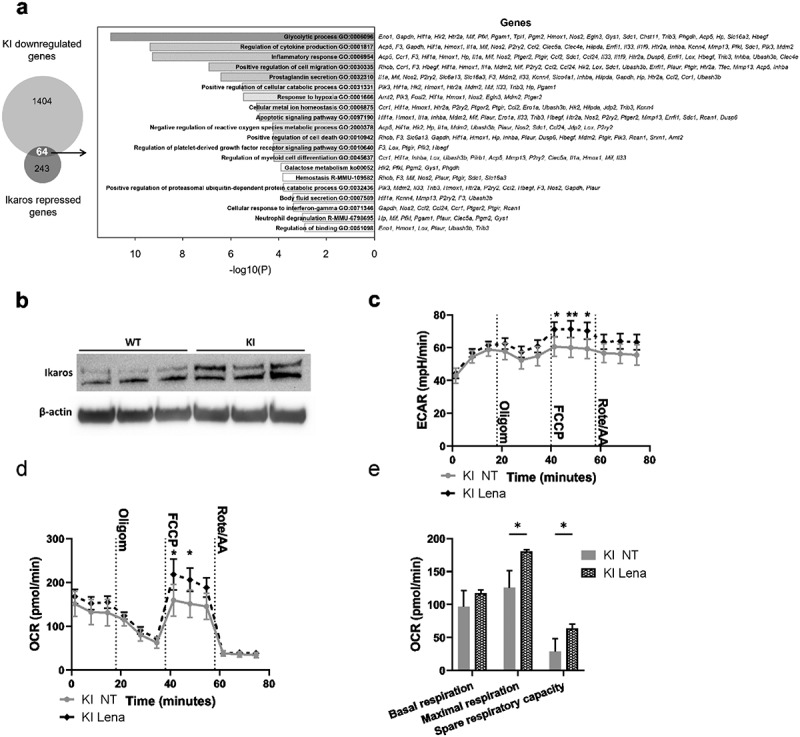
Transcription factor Ikaros has a key role in regulating β2-integrin-deficient BM-DC metabolism. (a) Comparison of genes repressed by Ikaros and genes being downregulated in β2-integrin KI BM-DCs. Here, the Ikaros-regulated genes are taken from^27^ and the genes being downregulated in β2-integrin KI cells are based on RNA-Seq expression data.^18^ GO-analysis result of the 64 intersect genes are also listed in a). (b) Ikaros levels in WT and β2-integrin KI BM-DCs was assessed by Western blotting (n=3). (c) ECAR and d) OCR in β2-integrin KI BM-DCs in the presence or absence of lenalidomide was assessed by Seahorse Extracellular Analyser with Mito Stress Test Kit as described in Materials and Methods (n=3). (e) Parameters of mitochondrial respiration were calculated based on OCR data shown in d). P-values are shown as <0.05*, <0.01**.

We have previously identified the transcription factor Ikaros (Ikzf1) as a major integrin-regulated factor involved in DC activation.^18^ Ikaros has been reported to function both as a transcriptional activator and a repressor, and to play a major role in DC development and function.^25,26^ Furthermore, our published data show that Ikaros plays a pivotal role in regulating a transcriptional network governing DC activation and migration in integrin-deficient cells.^18^ Therefore, to explore a putative role of Ikaros in regulating the metabolic phenotype of integrin-deficient DCs, we investigated the gene expression profile of β2-integrin KI BM-DCs and compared that to Ikaros repressed genes.^27^ Our bioinformatics analysis indicated an overlap between Ikaros-repressed genes^27^ and genes downregulated in β2-integrin KI BM-DCs (64 genes; Figure 5a). Interestingly, based on pathway analysis, genes involved in the glycolytic process scored the highest among the overlapping genes (Figure 5a). In addition, we found that the Ikaros levels were increased in β2-integrin KI BM-DCs compared to WT cells (Figure 5b). Therefore, to explore the functional role of Ikaros in mediating the integrin-regulated suppressed metabolic phenotype of BM-DCs, we utilized lenalidomide, an inhibitor of the Ikaros family transcription factors that leads to their selective proteasome-induced degradation.^28^ Indeed, the treatment of β2-integrin KI BM-DCs with lenalidomide increased the rates of both ECAR and OCR and thus reversed their metabolic phenotype (Figure 5c–e) but did not affect metabolism in WT cells (Supplementary SFig 1e). In particular, lenalidomide treatment led to upregulation of ECAR rates under conditions of metabolic stress (e.g. after FCCP treatment) in β2-integrin KI BM-DCs (Figure 5c). In addition, lenalidomide treatment led to increased oxidative phosphorylation, in particular, upregulation of maximal respiration and spare respiratory capacity in β2-integrin KI BM-DCs (Figure 5d–e, Supplementary Fig S1f). In contrast, lenalidomide did not significantly alter Glut1 expression or glucose uptake in KI cells (Supplementary Fig S1g-h), indicating that glucose uptake alone is not responsible for the Ikaros-regulated metabolic phenotype. Together, these results indicate a key role of this transcription factor in repressing the metabolic phenotype of integrin-deficient BM-DCs.

### Mimicking integrin-regulated metabolic suppression with low-dose 2DG activates BM-DCs through an epigenetic mechanism

**Figure 6.**
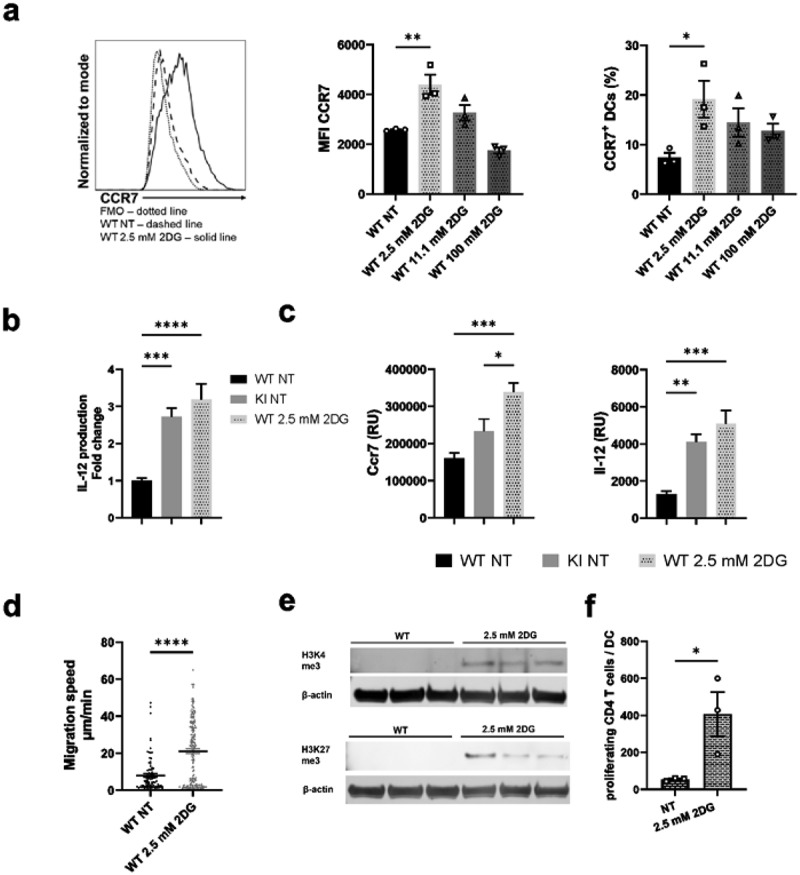
Inducing metabolic stress with low-dose 2DG activates BM-DCs. a) WT BM-DCs were treated with different amounts of 2DG, after which CCR7 expression was assessed by flow cytometry. Representative histograms for NT WT and 2.5 mM 2DG-treated WT BM-DCs, MFIs and proportion of CCR7 expressing cells are shown (n=3). b) Production of IL-12 by NT WT, NT β2-integrin KI or 2.5 mM 2DG-treated WT BM-DCs was assessed by ELISA (n=15–16). Data is pooled from five independent experiments. c) mRNA expression of CCR7 and IL-12 in NT WT, NT β2-integrin KI and 2.5 mM 2DG-treated WT BM-DCs, was assessed by qRT-PCR (n=5). d) Migration speed of WT BM-DCs with or without 2.5 mM 2DG-treatment in a 3D collagen matrix in response to a CCL19 gradient was assessed as described in the Materials and Methods (n=3 mice, a total of 267 WT and 346 2DG-treated WT cells were tracked). e) Histone methylation of H3K4me3 and H3K27me3 in NT WT and 2DG-treated WT BM-DCs was assessed by Western blotting (n=3). f) T cell proliferation was assessed by MLR by culturing CD4 T cells with NT or 2.5mM 2DG-treated BM-DCs. p-values are shown as <0.05*, <0.01**, <0.005***, <0.0001****.

Here, we show that BM-DCs expressing dysfunctional integrins display a suppressed metabolic phenotype. To explore whether such integrin-regulated metabolic suppression may contribute to DC activation, we investigated the consequences of WT BM-DCs treatment with various concentrations of 2-deoxy glucose (2DG), an inhibitor of glycolysis (Figure 6). 2DG is a glucose analog that competes with glucose by binding to hexokinase, the rate-limiting enzyme of glycolysis, but it also reduces oxidative phosphorylation in cells by affecting pyruvate levels that feed into the TCA cycle, fueling oxidative phosphorylation.^29^ Interestingly, metabolic suppression induced by treatment of WT BM-DCs with a low-dose of 2DG (2.5 mM) (in the presence of 11.1 mM glucose in the media) indeed led to their activation. Specifically, a low-dose 2DG treatment led to increased expression of the chemokine receptor CCR7 and production of the cytokine IL-12, both on the protein and mRNA levels (Figure 6a–c), whilst a higher dose of 2DG (11.1 mM), which blocks glycolysis and reduces oxidative phosphorylation in cells^29^ did not have this effect (Figure 6a). In addition, other low doses of 2DG, 1 mM and 5 mM, resulted in lower CCR7 expression and/or IL-12 production in WT BM-DCs compared to 2.5 mM 2DG-treatment, suggesting 2,5 mM being the optimal 2DG dose to activate BM-DCs (Supplementary Fig S2a). Low-dose (2,5 mM) 2DG-treatment also led to significantly increased BM-DC migration speed in a 3D collagen matrix in response to the chemokine CCL19 (Figure 6d). In contrast, 2.5 mM 2DG treatment did not affect the expression of CD40, CD86, CD80, or MHC-II in WT cells (Supplementary Fig S2b), indicating that all aspects of the activated DC phenotype are not affected by suppressed cell metabolism. 2.5 mM 2DG treatment led to a further increase in CCR7 expression and IL-12 production also in β2-integrin KI cells (Supplementary Fig S2c), showing that further suppression of metabolism can also activate KI cells. However, compared to WT cells, 2.5 mM 2DG-treatment did not significantly increase the proportion of cells expressing CCR7 in the β2-integrin KI population, whilst in WT cells the proportion was significantly increased (Supplementary Fig S2d). Together with the experiments showing that metabolism is significantly reduced in β2-integrin KI cells, this experiment shows that reduced metabolism downstream of integrins in DCs may contribute to their activation.

Interestingly, 2.5 mM 2DG-treatment also increased CCR7 expression in LPS-activated BM-DCs (Supplementary Fig S3a) and in LPS or CpG activated Flt3L-induced BM-DCs (Supplementary Fig S3b-c). Notably, in Flt3L-induced BM-DCs, CCR7 expression significantly increased in CpG-activated conventional DC type 1 (cDC1) population and also in CpG or LPS-activated conventional DC type 2 (cDC2) population following 2.5 mM 2DG-treatment (Supplementary Fig S3c). In addition, 2.5 mM 2DG-treatment also increased CCR7 expression in a recently characterized human DC cell line (CAL-1) (Supplementary Fig S3d), thereby expanding a potential role for metabolic suppression in DC activation also to other murine DC populations and to human cells. However, changing the metabolic rate of WT BM-DCs in other ways, such as by excluding glucose or glutamine from the medium (thereby reducing glycolysis and glutaminolysis), did not significantly change BM-DC CCR7 or IL-12 expression (Supplementary Fig S4a). Similarly, treating cells with varying concentrations of rotenone (to inhibit oxidative phosphorylation) did not lead to a significant increase in IL-12 production (Supplementary Fig S4b). Interestingly, treating WT BM-DCs with benserazide, an inhibitor of Hk2 (a rate limiting enzyme of the glycolytic pathway), led to increased proportion of cells expressing CCR7 but did not affect CCR7 MFI or IL-12 production (Supplementary Fig S4c). These results indicate that blocking glycolysis or oxidative phosphorylation alone did not result in BM-DC activation; rather, partial metabolic suppression (of both glycolysis and oxidative phosphorylation, by using low-dose 2DG) was required to achieve BM-DC activation.

We have previously shown that β2-integrins regulate BM-DC activation through an epigenetic mechanism (H3K4me3 and H3K27me3 on specific genes).^18^ Interestingly, low-dose 2DG-treatment of BM-DCs also increased both H3K4me3 and H3K27me3 levels in WT BM-DCs, indicating that suppressing glycolytic metabolism increases DC activation through an epigenetic effect (Figure 6e).

To investigate whether the low dose 2DG-induced increase in DC activation also correlates with T cell responses, we performed mixed lymphocyte reactions (MLR) of either non-treated (NT) or low dose 2DG-treated BM-DCs with T cells. Interestingly, T cell proliferation was significantly increased with 2.5 mM 2DG-treated DCs compared to NT DCs, showing the functional significance of metabolic suppression in DC phenotype (Figure 6f). In conclusion, metabolic suppression induced by low-dose 2DG-treatment activates BM-DCs in a similar way as integrin deficiency.

### Metabolic suppression of BM-DCs with low dose 2DG-treatment ex vivo improves their anti-tumor responses in vivo

**Figure 7.**
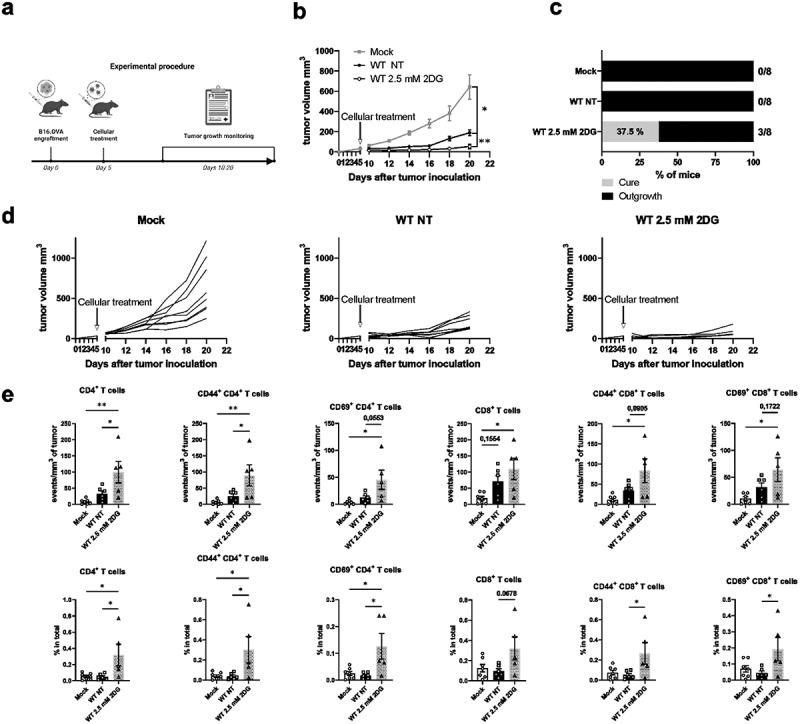
Metabolic suppression improves BM-DC-mediated anti-tumor responses in vivo. a) Experimental design of the tumor experiment. b) Growth of B16-OVA melanoma tumors before and after peritumoral injection of PBS (Mock), non-treated (NT) WT BM-DCs or WT BM-DCs treated with 2.5mM 2DG (n=8 mice per group). Tumor growth was measured every 2 d. c) the fractions of mice with tumor biomass (outgrowth) and no tumor biomass (cure) 20 d after tumor inoculation. d) Individual growth curves of B16-OVA melanoma tumors before and after peritumoral injection of PBS (Mock) (left panel), non-treated WT BM-DCs (middle panel) or WT BM-DCs treated with 2.5mM 2DG (right pane). e) Numbers and proportions of CD4 T cells, CD44^+^(memory) CD4 T cells, CD69^+^ (activated) CD4 T cells, CD8 T cells, CD44^+^ (memory) CD8 T cells and CD69+ (activated) CD8 T cells in tumors, relative to tumor volume were determined flow cytometrically.

Our data indicate that metabolic suppression contributes to BM-DC reprogramming to an active, migratory phenotype. We have previously shown that integrins and integrin-regulated BM-DC epigenetic reprogramming regulate BM-DC anti-tumor responses through a CCR7-dependent mechanism.^18^ As we show here that partially suppressing BM-DC glycolytic metabolism leads to BM-DC activation (increased cytokine responses, CCR7 expression, migration speed, and T cell activation potential), we decided to explore the effect of 2DG-induced metabolic stress in BM-DCs on tumor growth *in vivo*. For this, we used a well-established immunogenic mouse melanoma tumor model (B16-OVA). Following tumor establishment, WT mice were injected peritumorally either with PBS (Mock), with NT WT BM-DCs or with low-dose 2DG-treated WT BM-DCs, after which tumor growth was followed (Figure 7a). Interestingly, the treatment of tumor-bearing mice with 2DG-treated WT BM-DCs led to significantly reduced tumor growth compared to mice treated with non-treated WT BM-DCs (Figure 7b,d). Further, treatment with 2DG-treated WT BM-DCs also led to the elimination of tumors in a notable percentage of the mice (Figure 7c). Finally, even though the low-dose 2DG-treatment caused some decrease in BM-DC viability (Supplementary Fig S4d), 2DG-treated WT BM-DCs were still able to induce stronger anti-tumor response *in vivo* compared to NT WT BM-DCs.

P-values are shown as <0.05 *, <0.01 **.

Next, we performed immunophenotyping to the B16-OVA tumors harvested at the end of the experiment, to assess tumor infiltrating T cell populations. Overall, 2.5 mM 2DG BM-DC-treated tumors were more heavily infiltrated by CD45^+^ leukocytes compared to the NT DC-treated or Mock-treated tumors (Figure 7e, Supplementary Fig S4e). There was an increase in CD4 T cell numbers as well as their proportions in tumors treated with 2DG BM-DCs compared to NT BM-DCs (Figure 7e). There were also more CD4 cells expressing CD44 (a memory T cell marker) in the tumors treated with 2DG-BM-DCs compared to NT DCs (Figure 7e). In addition, the proportion of activated (CD69-expressing) CD4 and CD8 T cells in the tumors (of total cells) were both increased, compared to tumors from NT DC-treated mice (Figure 7e). In conclusion, metabolic suppression with low-dose 2DG leads to BM-DC activation and increased anti-tumor responses mimicking loss of integrin-mediated adhesion, thus indicating a functional role for metabolic suppression in BM-DCs.

## Discussion

Integrins are adhesion and mechanoreceptors that interact with the extracellular matrix, soluble proteins, or receptors on other cells using their extracellular domains and with cytoskeletal and signaling proteins on the intracellular side. Integrins allow cells to “read” the mechanical properties of the microenvironments they reside in, leading to the activation of mechano-signaling pathways, which can also affect gene expression.^30^ DCs downregulate integrin adhesion when they become activated by infectious signals and need to become migratory,^5,7,31,32^ and thus, migDCs *in vivo* express low levels of β2-integrins.^6^ We have previously shown that BM-DCs expressing dysfunctional β2-integrins display increased activation *in vitro* and *in vivo*, increased T-cell stimulation capabilities, and increased anti-tumor responses *in vivo*. This integrin-regulated elevated activation of BM-DCs was shown to involve epigenetic regulation (increased H3K4me3 and H3K27me3-levels) of a large number of genes, including those encoding for maturation markers (CD40, CD80, and CD86), cytokines (IL-12), and chemokine receptors (CCR7).

Here, we show that β2-integrin-mediated adhesion also extensively regulates BM-DC metabolic profile. Loss of adhesion leads to a significant downregulation of metabolic rate in BM-DCs. We show that this is associated with lower protein translation, reduced levels of cellular ATP (and ADP but not AMP), reduction in many glycolytic enzymes and glucose transporters, reduced glucose uptake, and ROS production. Cells therefore acquire a metabolically repressed state as a consequence of loss of cell adhesion. In addition to glycolysis and mitochondrial function, metabolomics studies revealed that several other pathways are also dysregulated, such as amino acid metabolism. Interestingly, we show that this integrin-regulated metabolic suppression is not regulated by the master metabolic regulator, mTOR but is at least partly controlled by the transcription factor Ikaros (Ikzf1), which we have previously shown to control integrin-regulated BM-DC activation.^18^ Indeed, loss of Ikaros has been reported to lead to increased integrin activity, cell adhesion, and signaling in B cells.^33^ Ikaros has also been reported to restrict glycolytic metabolism in B cells, by regulating glucose uptake and glycolytic signaling.^34^ Together, these and our studies therefore show that Ikaros plays a major role as a “metabolic gatekeeper” in immune cells, restricting glycolytic processes in these cells leading to major consequences for immune cell function.

What we show here is profoundly different to what has been reported to take place during DC activation through pattern recognition receptors (PRRs), leading to significantly increased aerobic glycolysis but suppressed oxidative phosphorylation through HIF-1α and iNOS/nitric oxide (NO) production (NO inhibiting oxidative phosphorylation).^9^ The cell activation-associated increase in glycolysis has been shown to support fatty acid synthesis required for endoplasmic reticulum growth and synthesis of costimulatory molecules, cytokines, and chemokines^9^ and for the generation of ATP when oxidative phosphorylation is downregulated. In addition, inhibiting glycolysis by using high levels of 2DG has previously been shown to block LPS-induced BM-DC activation and DC-mediated T cell activation.^35^ Further, glucose and glycolysis have also been shown to be important for CCR7 oligomerization in DCs and for DC migration.^36^ In contrast, in integrin-deficient cells, decreased cell adhesion leads to increased BM-DC activation and epigenetic changes, resulting in increased expression of DC activation markers, cytokines, and chemokine receptors,^7,18^ but also to reduced metabolic rates (this study). It is important to note that in our system, there is no PRR stimulation of the cells, only loss of adhesion leading to loss of interaction of the cells with their extracellular microenvironment.

We further show that restriction of glycolysis in WT BM-DCs by low-dose (2.5 mM) 2DG-treatment leads to a similar response as integrin deficiency. A low dose of 2DG is expected to reduce both the rates of glycolysis and oxidative phosphorylation in BM-DCs,^29^ but not to block these pathways completely. Thereby, low-dose 2DG induces a suppressed metabolic phenotype similar to that seen in integrin-deficient cells, whilst complete blocking of glycolysis or oxidative phosphorylation did not have the same effect. We show that the treatment of BM-DCs with low concentration of 2DG leads to increased expression of CCR7 and IL-12, increased 3D migration *in vitro*, enhanced T cell activation potential *in vitro*, and induced epigenetic changes (changes in histone methylation) in the cells. Importantly, low-dose 2DG-treated BM-DCs also induce enhanced anti-tumor responses *in vivo* leading to reduced tumor growth, increased number of cures, and increased T cell infiltration into tumors in a mouse melanoma model. These results indicate that (integrin-mediated) metabolic control of DCs is also functionally important.

Glucose has previously been shown to limit BM-DC responses but in the context of TLR activation (by LPS).^22^ After the initial high need of glucose, at later time points, glucose seems to restrict LPS-induced BM-DC responses and T cell activation, as replacement of glucose with galactose actually stimulates BM-DCs.^22^ These results indicate that sustained high glycolysis also represses TLR-induced BM-DC activation. We show here that 2.5 mM 2DG treatment also enhances CCR7 expression in LPS treated DCs, in line with these previously published results.

Trained immunity or innate immune memory refers to a process in which innate immune cells, including DCs, display long-term epigenetic and metabolic reprogramming upon several types of stimuli, such as PRRs. In addition, as a consequence of this reprogramming, innate immune cells display altered responses toward a secondary challenge. Hence, the term “innate immune memory” has been coined to describe these alterations.^20^ Interestingly, loss of adhesion results in epigenetic changes in BM-DCs (changes in H3K4me3 and H3K27me3 status).^18^ The cells also display increased responses to secondary challenges such as LPS.^7^ Here, we show that loss of adhesion also leads to a repressed metabolic phenotype of BM-DCs, which is functionally important. Our results therefore indicate that innate immune cells such as DCs display epigenetic and metabolic reprogramming upon loss of adhesion. We call this process “mechanical immune memory”. Loss of adhesion may be a “danger signal” which activates DCs and leads to reprogramming and altered responses to a secondary challenge. Mechanical disruption of DC cellular interactions has indeed previously been found to lead to profound DC activation, but the underlying mechanisms have remained poorly understood.^37^ We thus propose that mechanical interactions between tissue innate immune cells with their microenvironment have a bigger impact on immune responses than previously thought.

It is striking that treatment with low dose of 2DG, an inhibitor of glycolysis and oxidative phosphorylation, induces metabolic reprogramming of BM-DCs in a similar manner as integrin deficiency.^18^ Importantly, this DC activation caused by metabolic restriction is reflected in increased DC-mediated tumor growth inhibition *in vivo*, presumably by affecting CCR7-dependent DC migration to lymph nodes and/or T cell activation, where IL-12 production plays a major role, or a combination of these effects. We show here that 2.5 mM 2DG-treatment increases CCR7 expression also in Flt3L-induced BM-DCs (which more closely resemble *in vivo* cDCs compared to GM-CSF-induced DCs) and in a human DC cell line. In the future, it will be essential to investigate the effect of low-dose 2DG treatment (or similar) on human primary DCs. DC-based immunotherapies are promising as they are already in clinical use, considered to be safe and induce immune responses in at least half of the treated patients.^2^ However, their clinical efficacy is less impressive, and these therapies clearly need to be further optimized.^2^ For example, the migratory and T cell activation capabilities of current DC vaccines are still relatively poor.^2^ Based on the results presented here and previously,^18^ targeting integrins or integrin-regulated epigenetic or metabolic pathways may therefore be a useful way to optimize DC-based immunotherapeutic approaches to tumors in the future and could also be combinable with other immunotherapeutic approaches such as immune checkpoint inhibitors.

## Materials and methods

### Mice and bone marrow collection

Integrin TTT/AAA β2-integrin knock-in mice (β2-integrin KI) have been previously described.^38^ C57BL/6N WT littermates were purchased from Charles River and were used as controls for β2-integrin KI mice. Experiments were performed using male and female mice between ages 8 and 39 weeks. Animals were housed under conventional conditions in groups of up to five animals per cage with free access to food and water. Bone marrow was collected from euthanized animals and used for BM-DC cultures immediately. For the tumor experiment, C57BL/6J female mice were purchased from Charles River. Mice were 24 weeks old when the experiment started. All the experiments were performed according to the Finnish Act on the Protection of Animals Used for Scientific or Educational Purposes (497/2013) and to the Directive 2010/63/EU of the European Parliament and of the Council of 22 September 2010 on the protection of animals used for scientific purposes and approved by the Finnish National Animal Experiment Board (Hankelupalautakunta – ELLA).

### BM-DC culture

BM-DCs were generated by culturing bone marrow cells for 9–10 d in DC media: 10 ng/ml GM-CSF (Peprotech, cat. # AF-315-03) in RPMI (Lonza, cat. # 12-167F/EuroClone, cat. # ECB9006L) supplemented with 10% FCS (Gibco, cat # 10500–064), 100 U/ml penicillin–streptomycin (penicillin, Orion, cat. # 465161; streptomycin Thermo Fisher Scientific, cat. # D7253-100 g) and 2 mM L-glutamine (Thermo Fisher Scientific, cat. # BP379–100), at 37°C in a humidified atmosphere of 5% CO_2_. Media were added/changed on days 3, 6, and 8. In some experiments, cells were treated overnight with 20 nM rapamycin (Sigma-Aldrich, cat. # R8781-200UL), 10 µM Lenalidomide (Sigma-Aldrich, cat. # SML2283), 2DG (various concentrations) (Sigma-Aldrich, cat. # D8375), rotenone (various concentrations) (Sigma-Aldrich, cat. # R8875), glucose (various concentrations) (Sigma-Aldrich, cat. # G7021), L-glutamine (Thermo Fisher Scientific, cat. # BP379–100), benserazide (various concentrations) (Sigma-Aldrich, cat. # B7283) or 100 ng/ml LPS (Sigma-Aldrich, cat. # L6529). In glucose and glutamine exclusion experiments, cells were grown as above but overnight in RPMI media without glucose (MP Biomedicals, cat. # 091646854). To generate Fl3L-induced BM-DCs, bone marrow cells were cultured in 200 ng/ml Flt3L (Peprotech, cat. # 250-31 L) for 9 days, with feeding on days 3 and 6. On day 9, cells were stimulated overnight with 200 ng/ml Flt3L and either 1 µM CpG (InvivoGen, cat. # tlrl-1826) or 100 ng/ml LPS with or without 2.5 mM 2DG.

### Tumor models and cell lines

The ovalbumin-expressing murine melanoma cell line B16-OVA was provided by Prof. Richard Vile (Mayo Clinic, Rochester, MN, USA). Mycoplasma test was routinely done one-week post-culture by analyzing the supernatant of the cells with MycoAlertTM Mycoplasma Detection Kit (Lonza, cat. #: LT07). The cell line was cultured in RPMI medium (Lonza, cat. # 12-167F) supplemented with 10% FBS (Thermo Fisher Scientific, cat. # 10500–064), 2 mM L-glutamine (Thermo Fisher Scientific, cat. # BP379–100), 1% penicillin/streptomycin (penicillin, Orion cat. # 465161; streptomycin Thermo Fisher Scientific, cat. # D7253-100 g). B16-OVA was cultured under Geneticin (Thermo Fisher Scientific, cat. # 10131035) selection at 37°C in a humidified atmosphere of 5% CO_2_. The human DC cell line CAL-1^39^ was obtained from Leibniz-Institut Deutsche Sammlung von Mikroorganismen und Zellkulturen (DSMZ, #ACC923). Cells were cultured in a complete growth medium (RPMI 1640 supplemented with 10% FCS, 100 U/ml penicillin–streptomycin (penicillin, Orion, cat. # 465161; streptomycin Thermo Fisher Scientific, cat. # D7253-100 g) and 2 mM L-glutamine (Thermo Fisher Scientific, cat. # BP379–100)), at 37°C in a humidified atmosphere of 5% CO_2_. Cells were treated with 10 ng/ml human GM-CSF (Peprotech, cat. # 300–03) for 3 d and then cultured overnight without GM-CSF but with or without 2.5 mM 2DG.

### Seahorse analysis

Oxygen consumption rate (OCR) and extracellular acidification rate (ECAR) were measured with Seahorse XF96 extracellular analyzer (Agilent) by using Seahorse XF Cell Mito Stress Test Kit (Agilent, cat. # 103015–100). Briefly, BM-DCs were detached with 5 mM EDTA in PBS and 100 000 cells/well plated in DC media on Seahorse XF96 cell culture microplates (Agilent, cat. # 101085–004). Following an o/n incubation at 37°C, 5% CO_2_, the plates were centrifuged 300 G for 3 min at +4°C and DC media was replaced by pre-warmed Seahorse media (Seahorse XF RPMI [Agilent, cat. # 103576–100], supplemented with 10 mM glucose [Sigma-Aldrich, cat. # D9434], 5 mM pyruvate [Sigma-Aldrich, cat. # P8574] and 5 mM glutamine [Sigma-Aldrich, cat. # G3126] and adjusted to pH 7.4). After 1 h incubation at 37°C, avoid from CO_2_, the basal OCR and ECAR were measured, followed by sequential injection of oligomycin (1 μM) (Sigma-Aldrich, cat. # O4876), FCCP (1 μM) (Sigma-Aldrich, cat. # C2920), and rotenone/antimycin A (1 μM) (Sigma-Aldrich, cat. # R8875, A8678) at the indicated time points. Parameters such as basal respiration, maximal respiration, and ATP production were calculated according to manufacturer’s instructions (Agilent). Data analysis was done by using Wave software (Agilent) and finalized in Excel and GraphPad Prism. All experiments had ten technical replicates, and in some experiments, the inhibitor treatments were added for o/n incubation.

### Targeted LC-MS metabolomics profiling

BM-DCs were collected from petri dishes by scraping into ice-cold PBS and pelleted. Metabolites were extracted from cell pellets using 400 µl of ice-cold extraction solvent (Acetonitrile:Methanol:MQ; 40:40:20; Thermo Fisher Scientific, ACN cat. # A955–212, MeOH cat. # A456–212) and subsequently, samples were vortexed for 2 min, sonicated for 1 min followed by centrifugation at 14000 rpm at 4°C for 5 min. Supernatants were transferred into HPLC glass auto sampler vials (Thermo Fisher Scientific cat. # 11575894) and 2 µl of samples were injected into the Thermo Vanquish UHPLC coupled with Q-Exactive Orbitrap quadrupole mass spectrometer equipped with a heated electrospray ionization (H-ESI) source probe (Thermo Fisher Scientific) for analysis. The gradient elution was carried out with the following settings: flow rate of 0.100 ml/min using 20 mM ammonium hydrogen carbonate, adjusted to pH 9.4 with ammonium solution (25%) as mobile phase A and acetonitrile as mobile phase B. The gradient elution was initiated from 20% of mobile phase A and 80% of mobile phase B and maintained till 2 min, followed by 20% of mobile phase A gradually increasing up to 80% till 17 min, then 80% to 20% Mobile phase A decrease in 17.1 min and maintained up to 24 min. The column oven and auto-sampler temperatures were set to 40 ± 3°C and 5 ± 3°C, respectively. MS had the following settings: polarity switching, resolution of 35,000, the spray voltages of 4250 V for positive and 3250 V for negative mode, the sheath gas: 25 arbitrary units (AU), and the auxiliary gas: 15 AU, sweep gas flow 0, Capillary temperature: 275°C, S-lens RF level: 50.0. Instrument control was operated with the Xcalibur 4.1.31.9 software (Thermo Fisher Scientific), and the peak integration was done with the TraceFinder 4.1 software (Thermo Fisher Scientific) using confirmed retention times standardized with library kit (Merck, cat. # MSMLS-1EA). The data quality was monitored throughout the run using an in-house serum QC sample and interspersed throughout the run as every 10th sample. The metabolite data were checked for peak quality (poor chromatograph), % RSD, and carryover. The intensity peak area data was provided as an Excel spread sheet and further analyzed with the publicly available MetaboAnalyst platform.

### Glucose uptake

Glucose uptake was measured using Glucose Uptake-Glo™ Assay kit (Promega, cat. # J1341) following manufacturer’s protocol. Briefly, following BM-DC generation, the cells were detached and seeded on 96-well tissue culture treated with sterile ViewPlate microplate (PerkinElmer, cat. # 6005181) at 1 × 10^6^/ml concentration. Following an overnight incubation at 37°C in a humidified atmosphere of 5% CO_2_, the cells were washed with PBS (Lonza, cat. # 17-516F) and 1 mM 2DG (Sigma-Aldrich, cat. # D9761-100 MG) was added for 10 min. After stopping and neutralizing the 2DG uptake, 2-deoxyglucose-6-phosphate detection reagent was added for 2 h in dark RT. Luminescence was measured using Enspire™ Multimode plate reader and data analysis was done with GraphPad Prism. All reagents were included in the kit J1341 unless otherwise specified.

### Translation rate analysis

The translation rate of BM-DCs was measured through covalent incorporation of Puromycin (Gibco, cat. # A11138–03) into nascent proteins and assayed through flow cytometry using an anti-Puromycin PE conjugated antibody (BioLegend, cat. # 381503) after fixing and permeabilizing the cells using the Foxp3 permeabilization kit (eBiocience, cat. # 00-5523-00). As a negative control, translation was blocked by treating the cells with Harringtonine (Sigma-Aldrich, cat. # SML1091-10 MG) at 2 µg/ml for 30 min before the addition of Puromycin. Puromycin was added at 10 ug/ml for 45 min after 30 min of incubation with the inhibitor. The incorporation of puromycin was then assayed via flow cytometry.

### ROS measurements

The level of ROS was assessed with ROS-ID Total ROS/Superoxide Detection Kit (Enzo, cat. # ENZ-51010) according to the manufacturer’s protocol. Briefly, BM-DCs were first washed with a wash buffer and then resuspended in a wash buffer with a concentration of 2 × 10^6^ cells/ml. 5 × 10^5^ cells were transferred in flow cytometry tubes, mixed with equal amount of 2× staining solution and incubated coverage from light at +37°C for 30 min. Data were collected using an LSR Fortessa flow cytometer (Becton Dickinson) and analyzed with FlowJo (Tree Star).

### Mitochondrial membrane potential

The level of mitochondrial membrane potential was assessed by using MITO-ID Membrane Potential Detection Kit (Enzo, cat. # 51018) according to the manufacturer’s instructions. Briefly, 5 × 10^5^ BM-DCs per sample were first washed with an assay solution and then resuspended in a detection reagent and incubated coverage from light at RT for 15 min. Data were immediately collected following incubation using an LSR Fortessa flow cytometer (Becton Dickinson) and later analyzed with FlowJo (Tree Star).

### ADP/ATP ratio

ADP, ATP, and ADP/ATP ratios were determined with ADP/ATP Ratio Assay Kit (Sigma-Aldrich, cat. # MAK135) by following the manufacturer´s protocol. Shortly, BM-DCs were detached and transferred onto the assay microplate, sterile 96-well tissue culture-treated ViewPlate microplate (PerkinElmer cat. # 6005181) with 10 000 cells/well. Following an o/n incubation at 37°C, 5% CO_2_, culture medium was replaced with ATP reagent and luminescence read with luminometer (Enspire™ Multimode plate reader) after 1-min incubation at RT (RLU_A_). The microplate was then incubated at RT for 10 min followed by another luminescence read to determine the level of background (RLU_B_). ADP reagent was then added to the wells, and the luminescence was again read after 1-min incubation at RT (RLU_C_). ADP/ATP ratio was calculated using the formula: ADP/ATP ratio = (RLU_C_-RLU_B_)/RLU_A_.

### Flow cytometry

The following conjugated antibodies were used for flow cytometric analysis of BM-DCs (company, catalog number, and clones given in brackets): CCR7-PE (BioLegend, cat. # 120105, clone 4B12), MHCII-APC-eFluor780 (eBioscience, cat. # 47-5321-82, clone M5/114.15.2), CD80-APC (cat. # 17-0801-81, eBioscience, clone 16-10A1), CD40-PE (cat. # 124609, BioLegend, clone 3/23), CD86-FITC (cat. # 11-0862-85, BD Biosciences, clone GL1), Puromycin-PE (BioLegend, cat. # 381503, clone 2A4). The following conjugated antibodies were used for flow cytometric analysis to determine T cells and DCs in MLR: CD3-AF488 (Invitrogen, cat. # 53-0031-82, clone 145-2C11), CD4-PE-Cy7 (BioLegend, cat. # 100528, clone RM4–5), CD11c-APC-ef780 (BioLegend, cat. # 117310, clone: N418). The following conjugated antibodies were used in tumor immunophenotyping: CD3-Biotin (eBioscience, cat. #13-0031-82, clone 145-2C11), CD69-FITC (BioLegend, cat. # 104506, clone H1.2F3), NK1.1-FITC (Invitrogen, cat. # 48-5941-82, clone PK136), Streptavidin-V500 (BD Bioscience, cat. # 561419), CD45-BV650 (BD Bioscience, cat. # 563410, clone 30-F11), CD8a-APC-Cy7 (BioLegend, cat. # 100714, clone 53–6.7), CD44-PE (BD Bioscience, cat. # 553134, clone IM7), CD4-PE-Cy7 (BioLegend, cat. # 100528, clone RM4–5). The following conjugated antibodies were used for flow cytometric analysis of Flt3L-induced BM-DCs: CCR7-PE (BioLegend, cat. # 120105, clone 4B12), MHCII-APC-eFluor780 (eBioscience, cat. # 47-5321-82, clone M5/114.15.2), CD11b-APC (eBioscience, cat. # 17-0112-82, clone M1/70), CD11c-PE-Cy7 (eBioscience, cat. # 25-0114-81, clone N418), B220-FITC (Southern Biotech, cat. # 1665-02S, clone RA3-6B2). The following conjugated antibody was used to flow cytometrically assess CCR7 expression in CAL-1 cell line: CCR7-PE (eBioscience, cat. # 12-1979-42, clone 3D12). Fc-receptor block (BD Pharmingen, cat. # 553142 clone 2.4G2) was used in all experiments assessing mouse cells, and unstained and FMO controls were included in all panels. Propidium iodide (Sigma-Aldrich, cat. # P4864) or 7-AAD (eBioscience, cat. # 00-6993-50) was used to detect dead cells. Acquisition was performed on an LSR Fortessa flow cytometer (Becton Dickinson), and data were analyzed using FlowJo software (Tree Star).

### ELISA

The level of IL-12 from WT and β2-integrin KI BM-DC supernatants was assessed with Mouse IL-12/IL-23 p40 Allele-specific DuoSet ELISA kit according to the manufacturer’s instructions (R&D, cat. # DY499). Briefly, a 96-well maxisorp microplate (Thermo Fisher Scientific, cat. # 442404) was first coated with capture antibody o/n. The next day, the wells were washed and blocked with a reagent diluent (1% BSA [Biowest, cat. # P6154] in PBS [Lonza, cat. # 17-516F]) for a minimum of 1 h. The plate was then washed, and standards and samples were added. Following a 2 h incubation, the plate was washed and the detection antibody was added to the wells. After 2 h, the plate was washed, and Streptavidin-HRP (R&D, cat # DY998) was added for 20 min. After washing, the substrate solution (R&D, cat. # DY499) was added in the wells. Following visible color development (or latest after 20 min), sulfuric acid (Acros Organics, cat. # 124645001; diluted 1:4 in water) was added to stop the reaction. The optical density was immediately determined at 450 nm and background at 540 nm wavelength using Multiskan GO spectrophotometer (Thermo Fisher Scientific). IL-12 concentration was determined based on the standard curve, and data analysis was done using Excel and GraphPad Prism. All washing steps were performed by washing the plate first three times with 0.05% Tween (Fisher BioReagents, cat. # BP337) in PBS and then once with PBS only. All reagents were included in the kit DY499 unless stated otherwise.

### Western blotting

Cells were lysed in a cold M-PER lysis buffer (Thermo Fisher Scientific, cat. # 78501) in the presence of phosphatase and protease inhibitors (Thermo Fisher Scientific, cat. # A32961), and lysates were analyzed by standard Western blotting protocol using commercial gels (Invitrogen, cat. # NW04120) and nitrocellulose membrane (GE Healthcare, cat. # 10600004). Primary antibodies used were against H3K4me3 (Cell Signaling Technology, cat. #9751S), H3K27me3 (Cell Signaling Technology, cat. #9733S), GLUT1 (Millipore, cat. # 07–1401), p-AMPK (Cell signaling, cat. # 2535S), p-Akt (Cell signaling, cat. # 9275S), p-p70S6K (Cell signaling, cat. # 9234S), Akt (Cell signaling, cat. # 9272S), Ikaros (Santa Cruz, cat. # sc-398,265), and β-Actin (Cell signaling, cat. # 4970S). Quantification of the pAMPK blot was done by using Image Studio Lite (LI-COR).

### qRT-PCR

Total RNA was isolated from BM-DCs with Nucleospin RNA kit (Macherey-Nagel, cat. # 740955) and converted into cDNA by using High-Capacity cDNA Reverse Transcription kit (Thermo Fisher Scientific, cat. # 4368814) according to the manufacturer’s protocols. The qRT-PCR was performed by using TaqMan chemistry. Briefly, the cDNA was amplified in 11 µl volume containing TaqMan Fast Advanced Master Mix (Thermo Fisher Scientific, cat. # 4444557) and TaqMan primers/probes (Thermo Fisher Scientific cat. #;; Pfkl Mm00435587_m1; Pkm Mm00834102_gh; HIF1a Mm00468869_m1; Phd3 Mm00472200_m1; NOS2 Mm00440502_m1; CCR7 Mm01301785_m1; IL12 Mm00434174_m1). Each sample was run in triplicate, 18S rRNA (Thermo Fisher Scientific, cat. # 4333760T) was used as a reference gene, and no template control (NTC) was included in the assay. Reactions were run with CFX96 Touch Real-Time PCR Detection System (Bio-Rad) and data analyzed with CFX Maestro (Bio-Rad) and finalized with Excel (Microsoft) and Graph Pad Prism. Relative units were calculated by using the comparative CT (2^−ΔΔCt^) method.

### Mitochondrial DNA assay

Total DNA was extracted from BM-DCs using Nucleospin Triprep, Mini kit for RNA, DNA, and protein purification kit (Macherey-Nagel, cat. # 740966) according to the manufacturer’s instructions. The copy number of mitochondrial DNA was assessed using qRT-PCR. qRT-PCR reactions were run with CFX96 Touch Real-Time PCR Detection System (Bio-Rad) using Maxima SYBR Green/ROX qPCR Master Mix (Thermo Fisher Scientific, cat. # K0221). Each sample was run in duplicate, and *human β-globin* was used as a reference gene. Primers used in this study were as follows: mtDNA F: 5’-ACCACAGTTTCATGCCCATCGT-3’, mtDNA R: 5’-TTTATGGGCTTTGGTGAGGGAGGT-3’, β-globin F: 5’-GGTGAAGGCTCATGGCAAGAAAG-3’, and β-globin R: 5’-GTCACAGTGCAGCTCACTCAGT-3’. Data were analyzed with CFX Maestro (Bio-Rad) and finalized with Excel (Microsoft) and GraphPad Prism. Relative units were calculated by using the comparative CT (2^−ΔΔCt^) method.

### Cell migration assays

3D migration assays were performed with μ-Slide Chemotaxis 3D (Ibidi, cat. # 80326) imaging slides according to the manufacturer’s protocol. Briefly, WT BM-DCs were left untreated or treated with 2.5 mM 2DG overnight. Cells were mixed into a bovine collagen I (Advanced BioMatrix, cat. # 5005-B) and injected into the slide’s thin-imaging strip. After 45 min of collagen polymerization, one of the two chambers flanking the imaging strip was filled with media, the other with media containing mCCL19 (R&D systems, cat. # 361-MI-025) to a final concentration of 0.63 µg/µL. BM-DCs were imaged using the 3I Marianas imaging system (3I intelligent Imaging Innovations, Germany) by utilizing multipoint imaging. A 10×/0.30 EC Plan-Neofluar Ph1 WD = 5.2 M27 objective was used, and the dish was placed in a heated sample chamber (+37°C), in controlled 5% CO_2_ atmosphere. Cells were imaged using bright field microscopy. BM-DC migration was compared for 4 h by cell imaging every 1 min. Cells were tracked using the Manual Tracking (Fabrice Cordelières, Institut Curie, Orsay) plugin for ImageJ. All cells that started the timelapse in-frame were tracked for the duration of the timelapse or until they moved out of bounds of the image. The raw tracking data was fed into the Chemotaxis and Migration Tool (Ibidi) plugin for ImageJ. From there, we calculated the average speed of migration of each track and defined any track which moved faster than 1 μm/min over 4 h as a migrating BM-DC and used the speed of those tracks for further comparative analysis.

### Tumor experiment

B16-OVA cells were injected subcutaneously in the right flank (3 × 10^5^ cells/mouse), and the tumors were grown until palpable. Mice were then injected peritumorally with either 1 million untreated WT BM-DCs, 2DG-treated WT BM-DCs or PBS (MOCK). Eight mice per condition were used. Tumor growth was followed until the tumors reached the ethical size limit with a caliper every second day. Caliper measurements were done on both sides of the tumor and the volume was calculated by the following formula: V= (W × 2 × L), where W is the tumor width and L is the tumor length. At the end point, tumors were harvested and frozen at −80°C before assessing immune cell content by flow cytometry.

### Mixed lymphocyte reaction

CD4 T cells were isolated from the spleens of BALB/C mice with a CD4 T cell isolation kit (Miltenyi, cat. # 130-104-454) and labeled with cell-trace violet (Thermo Fisher Scientific, cat. # C34557) as per the manufacturer’s instructions. BM-DCs were pretreated with 2.5 mM 2-Deoxy-D-Glucose (Sigma-Aldrich, cat. # D8375-5 G) overnight or left untreated. For mixed lymphocyte reactions (MLRs), T cells and BM-DCs were resuspended in a complete medium (RPMI) supplemented with 10% FCS (Gibco, cat # 10500–064), 100 U/ml penicillin–streptomycin (penicillin, Orion, cat. # 465161; streptomycin Thermo Fisher Scientific, cat. # D7253-100 g), 2 mM L-glutamine (Thermo Fisher Scientific, cat. # BP379–100) and 50 µM 2-mercaptoethanol (Fluka biochemika, cat. # 63690). T cells (7.7 × 10^5^) were plated into 24-well tissue culture plate, and BM-DCs were added at 1:4 ratio. Cells were incubated for 5 d at 37°C and 5% CO_2_. T cell proliferation was measured by flow cytometry, by analyzing the number of T cells where cell trace violet was reduced as compared to the starting situation.

### Statistical analysis

Statistical significance between groups was assessed using t-tests or ANOVA followed by Tukey post-hoc. Mann–Whitney U-test was used for migration assay. All data represent mean ± SEM. All *p* values are shown as *, <0.05; **, <0.01; ***, <0.001; **** <0.0001.

## Supplementary Material

Supplemental Material

## Data Availability

Metabolomics data for this study were generated at the Institute for Molecular Medicine Finland (FIMM), University of Helsinki, Finland, and are available from the corresponding author upon request. All other data generated in this study are also available upon request from the corresponding author.
