# Supplementary material for: Loss of β2-integrin function results in metabolic reprogramming of dendritic cells, leading to increased dendritic cell functionality and anti-tumor responses
Source: Oncoimmunology. 2024 Jun 21;13(1):2369373. doi: 10.1080/2162402X.2024.2369373 (PMC11195491; doi:10.1080/2162402X.2024.2369373)
Supplement: Supplemental Material [file KONI_A_2369373_SM8736.docx]

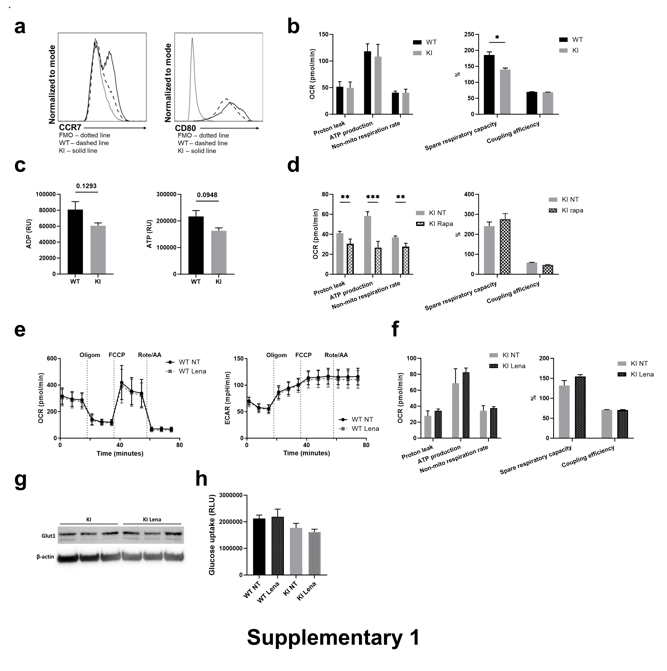


*Supplementary Fig 1. a) CCR7 and CD80 expression in WT and β2-integrin KI BM-DCs were measured by flow cytometry. Representative histograms are shown. b) Parameters of mitochondrial respiration in WT and β2-integrin KI BM-DCs were calculated based on OCR shown in Fig 2b (n=4). c) ATP and ADP concentrations in WT and β2-integrin KI BM-DCs were measured as described in Materials and Methods (n=3). d) Parameters of mitochondrial respiration in KI and rapamycin-treated KI BM-DCs were calculated based on OCR shown in Fig 4e (n=4). e)* *Extracellular acidification rate (ECAR) and oxygen consumption rate (OCR) of WT and lenalidomide-treated WT BM-DCs were measured real time by utilizing Seahorse Extracellular Analyser and Mito Stress Test Kit (n=4). f) Parameters of mitochondrial respiration in NT and lenalidomide-treated β2-integrin KI cells were calculated based on OCR shown in Fig 5d (n=3). g) Glut1 expression in NT and lenalidomide-treated β2-integrin KI cells was assessed by Western blotting (n=3). h) Glucose uptake in NT WT, lenalidomide-treated WT, NT β2-integrin KI and lenalidomide-treated β2-integrin KI cells was assessed by a glucose uptake kit, as described in Materials and Methods (n=3). P-values are shown as <0.05 *, <0.01 **, <0.005 ***.*


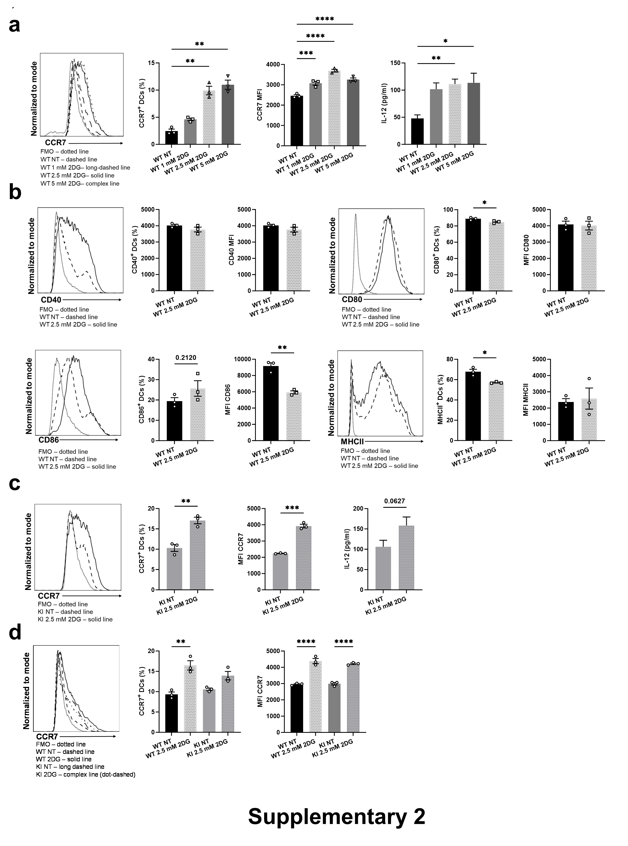


*Supplementary Fig 2. a) Effect of various concentrations of 2DG on CCR7 expression and IL-12 production in WT BM-DCs was measured by flow cytometry and ELISA, respectively (n=3). b) Effect of 2,5mM 2DG on expression of CD40, CD80, CD86 and MHCII in WT BM-DCs was assessed by flow cytometry (n=3). c) Effect of 2,5 mM 2DG on expression of CCR7 and IL-12 production in β2-integrin KI cells was assessed by flow cytometry (n=3) and ELISA (n=11), respectively. ELISA data is pooled from four independent experiments. d) Effect of 2,5 mM 2DG on CCR7 expression of WT and β2-integrin KI cells was assessed by flow cytometry (n=3). P-values are shown as <0.05 *, <0.01 **, <0.005 ***, <0.0001 ****.*


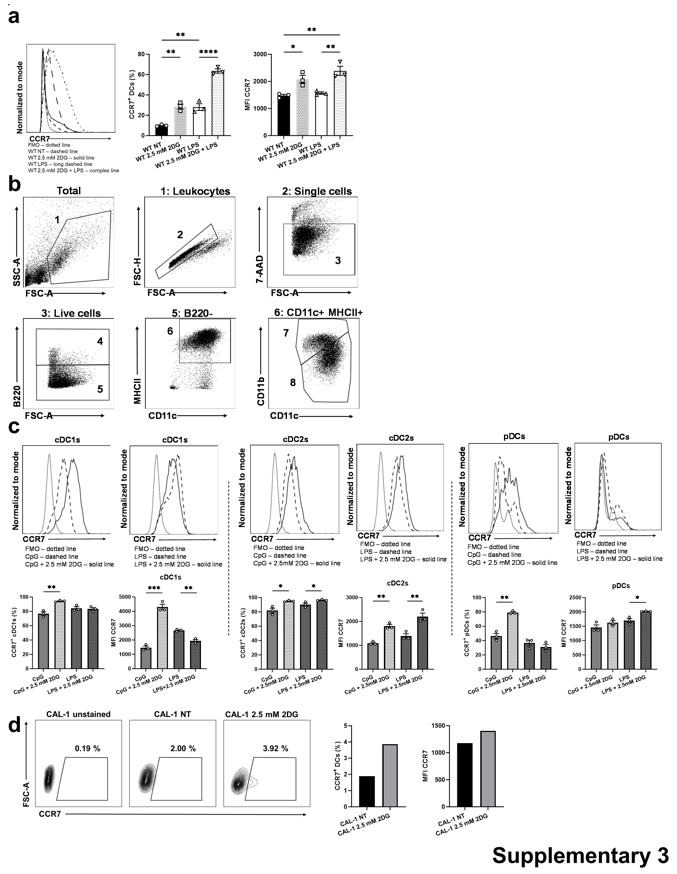


*Supplementary Fig 3. a) Effect of 2,5mM 2DG on expression of CCR7 in NT WT and LPS-treated WT BM-DCs was assessed by flow cytometry (n=3). b-c) Effect of 2,5 mM 2DG on CCR7 expression in Flt3L-induced BM-DCs was assessed by flow cytometry (n=3). Gating strategy is shown in b with gate 4 representing plasmacytoid DCs (pDCs), gate 7 conventional DCs type 2 (cDC2s) and gate 8 conventional DCs type 1 (cDC1s). Representative histograms and bar graphs for each cell population is shown in c. d) Effect of 2,5mM 2DG on CCR7 expression in the human DC cell line CAL-1 was assessed by flow cytometry (n=1). P-values are shown as <0.05 *, <0.01 **, <0.005 ***, <0.0001 ****.*


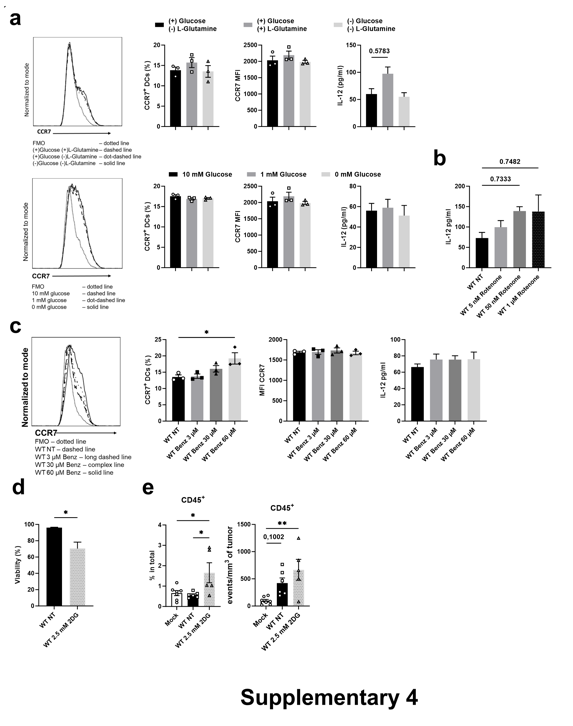


*Supplementary Fig 4. a) Effect of glucose and glutamine deprivation and also the effect of various concentrations of glucose on CCR7 expression and IL-12 production in WT BM-DCs was measured by flow cytometry and ELISA, respectively (n=3). b) Effect of varying concentrations of rotenone on IL-12 production was assessed by ELISA (n=3). c) Effect of varying concentrations of benserazide on CCR7 expression and IL-12 production in WT BM-DCs was assessed by flow cytometry (n=3) and ELISA (n=3-9), respectively. d) Viability of WT and 2.5mM 2DG-treated WT BM-DCs was assessed by Countess Automated Cell Counter (Invitrogen) (n=3). e) Proportion and number of CD45^+^ cells in tumors were determined flow cytometrically (n=5-7).* *P-values are shown as <0.05 *, <0.01 **.*
